# Multiobjective grammar-based genetic programming applied to the study of asthma and allergy epidemiology

**DOI:** 10.1186/s12859-018-2233-z

**Published:** 2018-06-26

**Authors:** Rafael V. Veiga, Helio J. C. Barbosa, Heder S. Bernardino, João M. Freitas, Caroline A. Feitosa, Sheila M. A. Matos, Neuza M. Alcântara-Neves, Maurício L. Barreto

**Affiliations:** 1 Center of Data and Knowledge Integration for Health (CIDACS), Instituto Gonçalo Muniz, Fundação Oswaldo Cruz, Salvador, Brazil; 2 Universidade Federal de Juiz de Fora, Juiz de Fora, Minas Gerais, Brazil; 3 Laboraório Nacional de Computação Científica, Petrópolis, Rio de Janeiro, Brazil; 4 Instituto de Saúde Coletiva, Universidade Federal da Bahia, Savador, Bahia, Brazil; 5 Instituto de Ciências da Saúde, Universidade Federal da Bahia, Savador, Bahia, Brazil

**Keywords:** Genetic programming, Asthma, Allergy, Classifier, Multiobjective

## Abstract

**Background:**

Asthma and allergies prevalence increased in recent decades, being a serious global health problem. They are complex diseases with strong contextual influence, so that the use of advanced machine learning tools such as genetic programming could be important for the understanding the causal mechanisms explaining those conditions. Here, we applied a multiobjective grammar-based genetic programming (MGGP) to a dataset composed by 1047 subjects. The dataset contains information on the environmental, psychosocial, socioeconomics, nutritional and infectious factors collected from participating children. The objective of this work is to generate models that explain the occurrence of asthma, and two markers of allergy: presence of IgE antibody against common allergens, and skin prick test positivity for common allergens (SPT).

**Results:**

The average of the accuracies of the models for asthma higher in MGGP than C4.5. IgE were higher in MGGP than in both, logistic regression and C4.5. MGGP had levels of accuracy similar to RF, but unlike RF, MGGP was able to generate models that were easy to interpret.

**Conclusions:**

MGGP has shown that infections, psychosocial, nutritional, hygiene, and socioeconomic factors may be related in such an intricate way, that could be hardly detected using traditional regression based epidemiological techniques. The algorithm MGGP was implemented in c ++ and is available on repository: http://bitbucket.org/ciml-ufjf/ciml-lib.

**Electronic supplementary material:**

The online version of this article (10.1186/s12859-018-2233-z) contains supplementary material, which is available to authorized users.

## Background

One of the major aims of epidemiology is to identify risk and protective factors associated with the occurrence of specific a diseases in humans. However, the study of these relationships in complex diseases, such as asthma and allergies, has proven to be difficult due to the large number of factors found to be related with these disorders [1]. Epidemiological studies very often use statistical tools like multivariate logistic regression and correlation analysis to model the relationships between risk factors and dichotomous outcomes [2]. While generally very powerful, these approaches assume that the predictive variables are independent and that the data can be modeled using linear combinations of these variables [3, 4]. As a consequence, when the relationships between these variables are nonlinear or they are interdependent (or conditionally dependent), the performance of the statistical approaches decreases. As many biological systems are fundamentally nonlinear and their parameters are conditionally dependent [4], the use of other approaches must be considered. In those situations, machine learning techniques has emerged as an useful alternative.

Machine learning is a branch of artificial intelligence that employs a variety of statistical, probabilistic and optimization techniques that allow computers to “learn” from past examples and to detect hard-to-discern patterns from large, noisy or complex systems. Machine learning has become a popular tool for medical researchers interested in predictive models, as well as, in the identification and exploration of patterns from biological phenomena. For instance, applications of machine learning techniques to different epidemiological problems can be found in the literature [4–7]. Many of these methods are efficient in modeling complex relationships between the independent variables. Unfortunately, these techniques often generate models that are difficult to interpret. Thus, the models generated by some machine learning approaches can be useless in clarifying the complex epidemiological relationships. Genetic programming [8] techniques in general, and the grammar-based [9] ones in particular, are exceptions as they are capable to generate interpretable models.

The theory of natural selection of Charles Darwin and Alfred Wallace has influenced much of human knowledge. The great ability of natural selection to generate biological complexity, efficiency of biological organisms, and adequate processes provided inspiration for the development of machine learning techniques such as the Genetic Algorithm. The Genetic Algorithm solves problems based on the process of natural selection. Genetic Programming is a particular type of Genetic Algorithm that can be used to generate computational artifacts (such as computer programs, mathematical models, logical models) that help explain observed data.

Grammar-based Genetic Programming is a specific type of genetic programming which uses a formal grammar that contains the rules and syntax used to generate appropriate solutions by the algorithm [9]. The use of a formal grammar to generate epidemiological models has two major advantages: (i) it enables the algorithm to generate more interpretable models by the use of a language closer to the human language, like the use of conditional relationships (if, else), logic (and, or) and comparatives (greater, lesser, equal); and (ii) it enables the researcher to establish his/her own rules for forming models and to introduce their knowledge in order to generate more appropriate models. Some studies have shown that grammatical genetic programming can be applied to several problems obtaining good results [10–12]

Multiobjective optimization problems (MOOP) are ubiquitous in real-world decision making. It is generally the case that a decision maker must simultaneously account for multiple criteria, with each criterion contributing to different objective to be optimized. Solving an MOOP involves obtaining a set of solutions that provide optimal tradeoff among all the relevant objectives constrain a Pareto-optimal solutions. In other words, a solution is considered optimal in the multiobjective sense if an attempted improvement in any one of its objectives is necessarily accompanied by the deterioration of at least one other objective [13].

The choice of an epidemiological model can be considered an MOOP because we can consider two criteria for selecting the best model: (i) the choice of more accurate models; (ii) the choice of models with reduced complexity (and thus more parsimonious). The Nondominated Sorting Genetic Algorithm II (NSGA) [14] applied in this study is a type of MOOP that uses the concept of dominance and the distances between the solutions for establishing the set of best solutions.

Asthma is among the most common chronic diseases worldwide, causing high levels of morbidity [15]. It is a heterogeneous condition with different phenotypes. It has been causally associated with diverse environmental factors as well as genetic backgrounds [16]. The prevalence of allergy and asthma has increased in affluent countries over recent decades, and has increased also in cities of non-affluent countries such as in Latin America [16, 17]. Such temporal trends occurring over a relatively short period of time are unlikely to be explained by changes in genetic susceptibility and are most likely explained by changes in environmental exposures such as those associated with the adoption of a modern or “westernized” lifestyle [18]. There are many studies showing different factors related to these disorders such as environmental factors [19, 20], socioeconomic [20, 21], infections [22–25], nutritional [26, 27], psychosocial [28, 29] and genetic [30, 31]. Recently, evidence has emerged to suggest that asthma causation may involve interactions between different exposures [30, 32]. Thus asthma and allergies can benefit from the use of techniques able to identify complex relationships.

This study evaluates the use of Multiobjective Grammatical Genetic Programming (MGGP) to find relationships between environmental, socioeconomic, infections, psychosocial and nutritional factors that may be related to the occurrence of asthma and allergies. To our knowledge, this methodology has not been applied before to solve an epidemiological condrum. We believe this approach could be of great use also in many other epidemiological problems lacking advanced tools for analyzing large and complex causal relationships.

## Methods

This section describes the study population, how asthma states were defined, allergy markers and genetic programming technique. This section also describes the techniques and methodologies applied in obtaining the data used to search for relationships between various exposures with the occurence of asthma and allergies. The expositions studied in this work cover anthropometric, psychosocial, diet, environmental and infections aspects.

### Study population and data collection

The study was a post hoc analysis of data collected during a survey of 1445 children aged 4-11 years and living in 24 poor neighborhoods in the city of Salvador, Northeast Brazil, performed in 2005 as part of a cohort study to investigate risk factors for asthma and allergy, and is described in detail elsewhere [33]. The neighborhoods and the children were selected as part of a previous study designed to measure the impact of sanitation on diarrhea [34]. Data on asthma symptoms were collected using a Portuguese-adapted ISAAC Phase II questionnaire, also a psychosocial and nutritional questionnaire was applied. The following measurements were performed for each child: anthropometric measurements, SPT testing and serum IgE for four aeroallergens, circulating IgG against six different pathogens, stool examination for detection of intestinal helminthic infections. The presence of mold on household walls was determined by direct inspection.

### Anthropometric measurements

The children were weighed on portable electronic scales (Filizola®;, model E-150/3P, with a capacity of 150 kg and accuracy of 100 g) and height was measured using stadiometers (Leicester Height Measure). Each measurement was done twice by different examiners and variations of 100 g for weight and 0.1 cm for height were accepted with the mean of duplicate observations used for calculation of body mass index (BMI) (weight[kg]/height[m]^2^). Z scores for BMI by age and gender were calculated against WHO 2006 reference values. Children with z-scores greater than 1.0 were considered to be overweight or obese [35, 36]. Previous studies carried out in Salvador indicated that overweight or obese may have important rule in development of asthma and allergy [37].

### Psychological disorder in the mother

The SRQ-20 questionnaire was used to assess minor psychiatric disorders in the mother. This instrument was developed by the World Health Organization [38] and validated in Brazil by Mari and Williams [39]. It is composed of 20 questions with dichotomous (yes/no) answers referring to the presence or absence of symptoms of depression, anxiety and somatic disorders in the previous month. A cut-off point for the definition of suspected cases of minor psychiatric disorders was established as 8 or more positive answers, a condition that, although not characterizing a psychiatric diagnosis, indicates significant psychic suffering. This cut-off point was defined in accordance with studies previously carried out in Brazil [39]. Also, previous studies carried out in Salvador reported an important association between minor psychiatric disorders in the mother and asthma symptoms in the child [40, 41].

### Dietary patterns

Information about the dietary patterns were obtained based on questionnaire of food frequency, validated by [42]. This questionnaire consists of 98 foods, related to food consumption in the last 12 months. A principal components analysis was used to obtain four food patterns that were represented by the numbers 1-4, and their value was discretized by their tertiles to create 4 levels. Details analysis to define the dietary pattern can be found in the work of [43]. The pattern 1 was characterized by the predominance of fruits, vegetables, legumes, cereals and fish. The pattern 2 was characterized by the predominance of milk and dairy products, ketchup / mayonnaise / mustard and chicken. The pattern 3 was characterized by the predominance of fried foods, sweets, snacks, coolant / artificial juice. The pattern 4 was characterized by the predominance of sausages, eggs and red meat.

### Allergen SPTs

SPTs were performed by two trained technicians using a standardized protocol and extracts of *D. pteronyssinus*, *B. tropicalis*, *B. germanica*, *P. americana*, dog and cat epithelia, and a fungal allergen mix (ALK-Abelló, São Paulo, Brazil). Extracts, saline and histamine controls were pricked onto the forearm skin using a disposable lancet (ALK-lancet^®;^; ALK-Abelló, São Paulo, Brazil). Reactions were read after 15 minutes and a reaction was considered positive if the mean diameter of the wheal was 3*mm* or larger than the saline control wheal. Frequencies of positive skin test reactions to dog and cat epithelia and a fungal allergen mix were low (< 4*%*) and were excluded from further analysis.

### Detection of intestinal helminth ova in fecal samples

Two fecal samples were collected two days apart and analyzed using the Hoffman sedimentation method and the Kato-Katz thick-smear technique [44] for the presence of helminth parasites (Trichuris trichiura, Ascaris lumbricoides, hookworms and Schistosoma mansoni). Hookworms and S. mansoni infections were rare (< 1%) and were not considered further in this analysis.

### Serum immunoassay for IgG to bacteria, protozoa, and viruses

Serum IgG antibodies to *Helicobacter pylori*, *Toxoplasma gondii*, herpes simplex virus (HSV), herpes zoster virus (HZV), Epstein-Barr virus (EBV) were measured using commercial ELISA kits (Diamedix, Miami, Florida, USA; Adaltis, Toronto, Canada). For the hepatitis A virus (HAV), kits from ADALTIS were used (Toronto, Canada). The assays were performed following the manufacturer’s instructions.

### Detection of allergen specific IgE by Immunocap

IgEs reacting with *Dermatophagoides pteronyssinus*, *Blomia tropicalis*, *Blatella germanica* and *Periplaneta americana* were measured in sera, using the Immunocap System (Pharmacia AB, Uppsala, Sweden), according to the manufacturer‘s instructions. Sera containing 0.70*kUIgE*/*L* or more were considered positive.

### Genetic Programming (GP)

Genetic programming (GP) is a special type of genetic algorithm which creates computational artifacts (for instance, computer programs written in a given language) to perform a given task. Although GP as it is known nowadays starts with Cramer [45], it was the work by Koza [8] that defined and popularized the method which was subsequently known as “standard GP”. In GP, the candidate solutions are referred to as “programs”, a high-level structure able to represent a large class of computational artifacts, such as a standard computer program, a numerical function or a classifier in symbolic form.

A population of candidate solutions is improved in GP following the same steps of a genetic algorithm. The steps of these techniques are presented in Algorithm 1 where “createInitialPopulation” creates the initial population, “evaluatePopulation” finds out how well the candidate solutions perform, “selectFittest” selects the best solutions with respect to their fitness, “crossover” combines each pair of parents generating new candidate solutions which are then mutated in “mutation”, and “replace” generates a new population by combining candidate solutions from the current(parent) and the offspring populations [46].


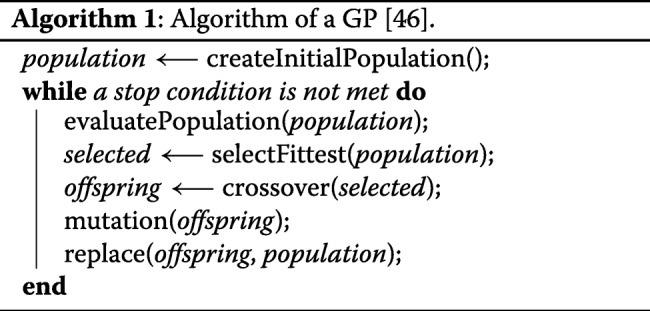


Typically, GP differs from standard genetic algorithm (i) in its representation of the candidate solutions, whereas GAs are intended to find an array of characters or numbers for representing the solution of a given problem, the goal of a GP process is to produce a “program” (or, as in our case, an expression) solving the optimization problem at hand, and (ii) in the definition of the move operators: crossover and mutation. The different possible ways of representing a candidate solution (such as trees, graphs, etc.) can be used to classify the GP variants.

Genetic programming has been applied to find solutions from a wide variety of fields. Producing patents and about 76 results that equals or surpasses the solutions found by human experts in their fields of research [47]. Among the fields of research we can highlight development of electric and quantum circuits [48], development of communication antennas [49], finite algebra [50], image recognition [51], symbolic regression [52] and reverse engineering [53].

In the study of biological systems the GP has been little applied, however recently several works have applied GP in the study of gene expression [54, 55], modeling of algal growth [56], prediction of cancer [57, 58], prediction of medical diagnosis [59], in the identification and classification of different types of scoliosis [60] and one area that GP has attracted interest is genome-wide association studies [61].

#### Grammar guided GP

Grammar guided GP [62], or grammar-based GP, uses grammars as a way to constrain the representation of the candidate solutions. Grammars can be used to create structures which belong to a specific language, and a formal grammar *G* can be defined as [63]

1 $$ G = \{N, \Sigma, R, S\},  $$

where *N* is a finite set of nonterminals (each nonterminal is formally delimited by < and >), *Σ* is a finite set of terminals or token symbols which are items that can appear in the language (such as constants, variables, and functions), *S*∈*N* is the start symbol, and *R* is a finite set of rules (or productions) which are as

2 $$ \left(\Sigma \cup N \right)^{\ast} N \left(\Sigma \cup N \right)^{\ast} ::= \left(\Sigma \cup N \right)^{\ast},  $$

where ^∗^ is the Kleene star operator^1^, ∪ denotes set union.

Typically, Grammar guided GP techniques use context-free grammars, a type of grammar in which the left-hand side of each production rule consists of a single nonterminal symbol, that is,

3 $$ N ::= \left(\Sigma \cup N \right)^{\ast}.  $$

**Fig. 1:**
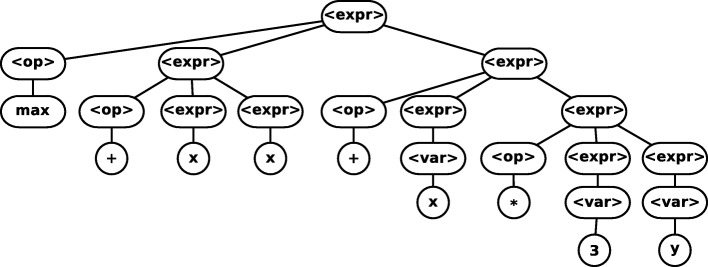
Example of a derivation tree [46]

The candidate programs in Grammar guided GP are represented by derivation trees, in which the internal nodes are the nonterminals of the grammar and the leaf nodes are symbols which appear in the language (terminals). An example of a derivation is available in the Fig. 1.

**Fig. 2:**
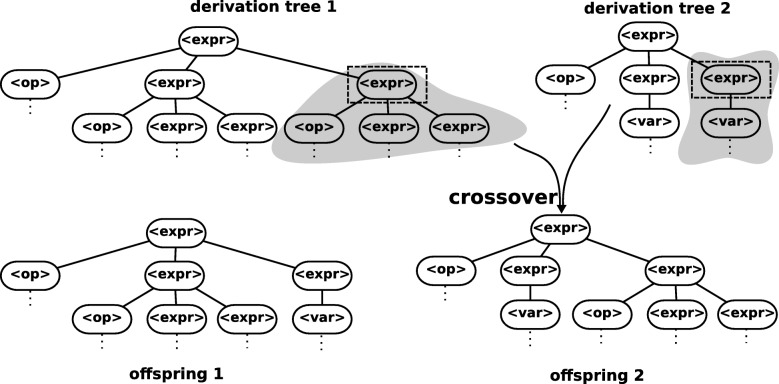
Example of crossover operators of Grammar Guided GP [46]

**Fig. 3:**
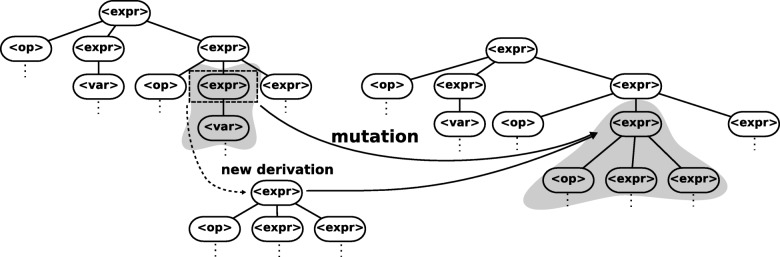
Example of mutation operators of Grammar Guided GP [46]

Grammar guided GP uses a grammar to guide the allowed representation of the candidate programs. The use of grammar delimits the creation of the initial population as well as the application of the variational operators as mutation and recombination. For both mutation and recombination, it is only permissible to exchange a non-terminal *N* for another of the same type, thus maintaining the consistency of the models. The recombination operator is shown in Fig. 2. It is randomly selected a non-terminal that exists in both parents and occurs the exchange of subtrees between parents. The mutation operator is shown in Fig. 3, a randomly selected subtree is replaced by another randomly created with the same non-terminal as root.

### Multiobjective Grammatical Genetic Programming (MGGP)

**Fig. 4:**
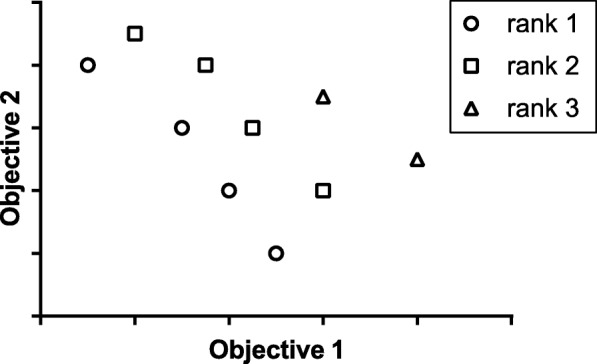
Example of domination rank with two objective, the rank 1 is nondominated, rank 2 is only dominated by rank1 and rank 3 is dominated by rank1 an rank 2
- The crowding distance computation requires sorting the population according to each objective function value. Thereafter, for each objective function, the boundary solutions (solutions with smallest and largest function values) are assigned as an infinite crowding distance value. All other intermediate solutions are assigned a distance value equal to the absolute normalized difference in the function values of two adjacent solutions. This calculation is continued with other objective functions. The overall crowding distance value is calculated as the sum of individual distance values corresponding to each objective. Each objective function is normalized before calculating the crowding distance.

An optimization problem seeks to find a solution that maximizes or minimizes an objective. However, many problems require finding the best solutions according to multiple objectives, thus being a multiobjective optimization problems (MOOP). The search for the relationships between factors associated with complex diseases such as asthma can be studied as a MOOP, where it is aim to maximize the accuracy and minimize the complexity of the relations simutaneously. This multiobjective approach aims to find the models that best explain this pathology being as simple as possible and therefore more parsimonious. The Grammar guided GP usually is applied to a mono-objective problem. To create the capability to solve MOOP, instead of using the obtained value of the objective function as criterion for selecting the best solutions in mono-objective problem. The MOOP algorithm NSGA [14] is based on dominance idea. Where one solution dominates the other if this solution is better in relation to all objectives, otherwise the solution is non-dominated. The NSGA uses two criteria for selecting the best solutions based on the objective functions:

- The dominance rank. All solutions which there is no other solution that is better than it for all objective functions simultaneously is call a nondominated solution. The rank 1 is formed by all nondominated solutions, rank 2 is formed for all solutions that are dominated by only rank1, and so on. This idea is illustrated in Fig. 4.

### Computational experiments

This section describes the details regarding the the computational experiments methodology. Firstly, it explains how the variables were chosen for modeling and then described as the preparation of the data with the formations of the study groups.

#### Model construction

Models were created for the explanation of 3 outcomes: Asthma, SPT and IgE against allergens. The exposure variables chosen were those that potentially represent the aspects that may be related directly or indirectly with asthma and allergy. The exposure variables and their frequency can be seen in Table 1 and were: gender, age, parental asthma, number of siblings, body mass index (BMI), HSV, HZV, EBV, HAV, *T. gondii*, *H. pylori*, *A. lumbricoides*, *T. trichiura* infections, four dietary patterns, daily calories, gross national income (GNI), mother psychological disorder, daycare ever, smokers at home, sewage disposal system, linen bed exchange, cat at home, dog at home, the presence of mold or moisture, piped water system, fly at home, paving of the street.

**Table 1:** Variables used to build Models

Variables	Type	Freq %
*N*=1046	Target variables	
IgE (positives)	Boolean	38.6*%*
SPT(positives)	Boolean	30.3*%*
Asthma (positives)	Boolean	22.9*%*
	Input variables	
Gender (males)	Boolean	52.7*%*
Age	Categorical	
4 and 5		35.9*%*
6 and 7		35.1*%*
8 to 11		29.0*%*
Parental asthma (presence)	Boolean	12.6*%*
HSV (positives)	Boolean	54.9*%*
HZV (positives)	Boolean	45.8*%*
EBV (positives)	Boolean	88.4*%*
HAV (positives)	Boolean	16.7*%*
*T. gondii* (positives)	Boolean	18.4*%*
*H. pylori* (positives)	Boolean	27.6*%*
*A. lumbricoides* (positives)	Boolean	16.2*%*
*T. trichiura* (positives)	Boolean	11.2*%*
Sibling number	Categorical	
none		18.9*%*
1		35.2*%*
2		24.0*%*
3 or more		21.9*%*
Daycare ever (yes)	Boolean	15.4*%*
Smoke at home (presence)	Boolean	27.1*%*
Sewage disposal system (presence)	Boolean	83.5*%*
Change bed linen ≥ 1 per week	Boolean	45.0*%*
Cat at home (presence)	Boolean	17.6*%*
Dog at home (presence)	Boolean	39.8*%*
Mold/moisture at home (presence)	Boolean	68.6*%*
Piped water system (presence)	Boolean	91.9*%*
Paving of the street (absence)	Boolean	35.1*%*
Fly at home (presence)	Boolean	51.5*%*
Mother Psychological disorder		
(suspect)	Boolean	37.2*%*
Dietary patterns 1 to 4	Categorical	Split by tertiles
Daily calories (*Kcalmean(sd)*)	Numerical	2210(929)
BMI	Categorical	
Overweight / Obesity		12.2*%*
Eutrophic		75.1*%*
Slimness		12.7*%*
GNI	Categorical	Split by tertiles

#### Data preparation

Most of the children who had missing data were due to refusal to withdraw blood samples, consequently they has missing for all serological data, or failing to provide all stool samples which made them missing all parasitological variables. This made it difficult to apply a methodology for imputation missing data. The use of individuals with missing data in the analyzes would cause different models to present different number of instances, which would compromise their adequate evaluation. We prefer to exclude all children who had missing data for any of the variables studied were excluded from the study then from the original 1445 children, 1047 has complete data. For realization of computational experiments the population was divided into groups. We randomly selected 10% of individuals (instances) to form the test group. This draw was made keeping the frequency of the outcome in the group equal to the frequency of the same in the original population. Of the remaining 90%, it was performed 6 times cross-validation where the population was randomly divided into 5 parts, maintaining the proportions in relation to the outcome equivalent to that of the original population. The first part of the population is defined as validation group and its respective training group consists of the other 4 parts. The process continue for for each part been validation group and the other ones been their respective training group. for each cross-validation was produced 5 validation group and their respective training group. At the end of the run 6 times the cross-validation was obtained 30 training groups with 751 subjects, their 30 validation groups with 190 subjects and one group with 105 subjects, respectively. All groups have relative frequencies similar to the original population. The same groups were used in all analyzes.

The study population showed more negative individuals for asthma and allergies than positive individuals (unbalanced database), and we therefore applied random over-sampling [64] in each training and validation group in order to prevent the negative group for asthma and allergies from having a greater influence on the accuracy than the positive group. Random over-sampling technique was not applied to test group.

#### MGGP

The MGGP was executed in 30 independent times for each training group. The MGGP was applied according to the standard algorithm for GP shown in the Algorithm 1. An initial population of 500 candidate solutions was randomly generated. The population was evaluated using NSGA based on two objective functions, (i) minimizing the classification error of the model in the training group (ii) minimizing the complexity of the model, given by the number of terminals in the tree representation of the candidate solution. The selection of parental solutions was carried out using tournament: two solutions were randomly selected and the best one of them was chosen to be a parent solution. Then the combination of two parental solutions generate two offspring solutions which suffer crossing and mutation. This process was repeated until 500 offspring solutions were generated and evaluated. The 500 best solutions between parent and offspring solutions were selected to form the next generation. The MGGP executed a total of 20,000 generations to obtain the final population. From the population of solutions at the end of the 20,000 generations, the solutions chosen as best were those that were non-dominated using the error in the validation group instead of the error in the training group to avoid problems with overfitting.

The context-free grammar built for this work contains comparison operators (>, <, >=, <=, == and !=), logic operators (*and*, *or*, *xor*) and the ternary operator *if-then-else*. To be easier to handle the solution computationally, it was used postfix notation. The grammar was composed by the following rules (*R*):

**Table:** 

<*expr*>::=	< *expr*1 > < *expr*1 > < *bool* > *if* − *else* | < *binaryClass* >
<*expr*1>::=	< *expr*2 > < *expr*2 > < *bool* > *if* − *else* | < *binaryClass* >
<*expr*2>::=	< *binaryClass* > < *binaryClass* > < *bool* > *if* − *else* |
	<*binaryClass*>
<*bool*>::=	<*varBin*> <*binaryClass*> <*compbin*> |
	<*varCat*> <*CatClass*> <*compcat*> |
	<*varNum*> <*numValue*> <*compCont*> |
	<*bool*> <*bool*> <*log*>
<*compcat*>::=	< | <= | == | >= | > | !=
<*compbin*>::=	== | !=
<*compCont*>::=	< | <= | > | >=
<*log*>::=	*and* | *or* | *xor*
<*binaryClass*>::=	0 | 1

where the symbol “ |” was used to delimit multiple derivation possibilities, “ <*varBin*>” were the set of binary variables, “ <*varCat*>” were the set of categorical variables, “ <*CatClass*>” were the possible values of each of categorical variables,“ <*varNum*>” were the set of numerical variables, and “ <*numValue*>” were the possible values of each of numerical variables. As we can observe, the rules presented for this work limit the number of nested *if-then-else* operations to 3 levels. This is to prevent the application of a new operation on a very small and non-representative group in terms of number of individuals.

#### Multivariate logistic regression (RL)

The RL models were generated for each of the training groups and then these generated models were evaluated on their respective validation and testing groups. For choice of exposure variables, the gender and age variables were considered a priori variables and always in the RL models The choice of other variables was by stepwise bidirectional selection [65] keeping the significant variables (*p-value*<0.05) in the model. As we want to compare a regression with classification models, the RL has been converted into a classification model by applying a step function on the predicted value, meaning that if the value predicted by the model is greater than 0.5 then the predicted value is 1, otherwise it is 0. These analyses were performed in Weka V3.6 2.

#### Classification algorithm C4.5

Models using the classification algorithm C4.5 [66], were also generated for each of the training groups and then were evaluated on their respective validation and testing groups. To avoid overfitting, the parameter of minimum number of instances per leaf was set to maximize the mean accuracy of the models for all executions in the validation groups. These analyses were performed in Weka. J48 is the Java implementation of C4.5 in Weka tool.

#### Classification algorithm random forest (RF)

The RF [67] algorithm was applied in the 30 training groups. The parameter maximum size of the trees chosen was 3, because this presente the smallest errors in the validation group after the models be generated in the training groups. These analyses were performed in Weka.

## Results

**Fig. 5:**
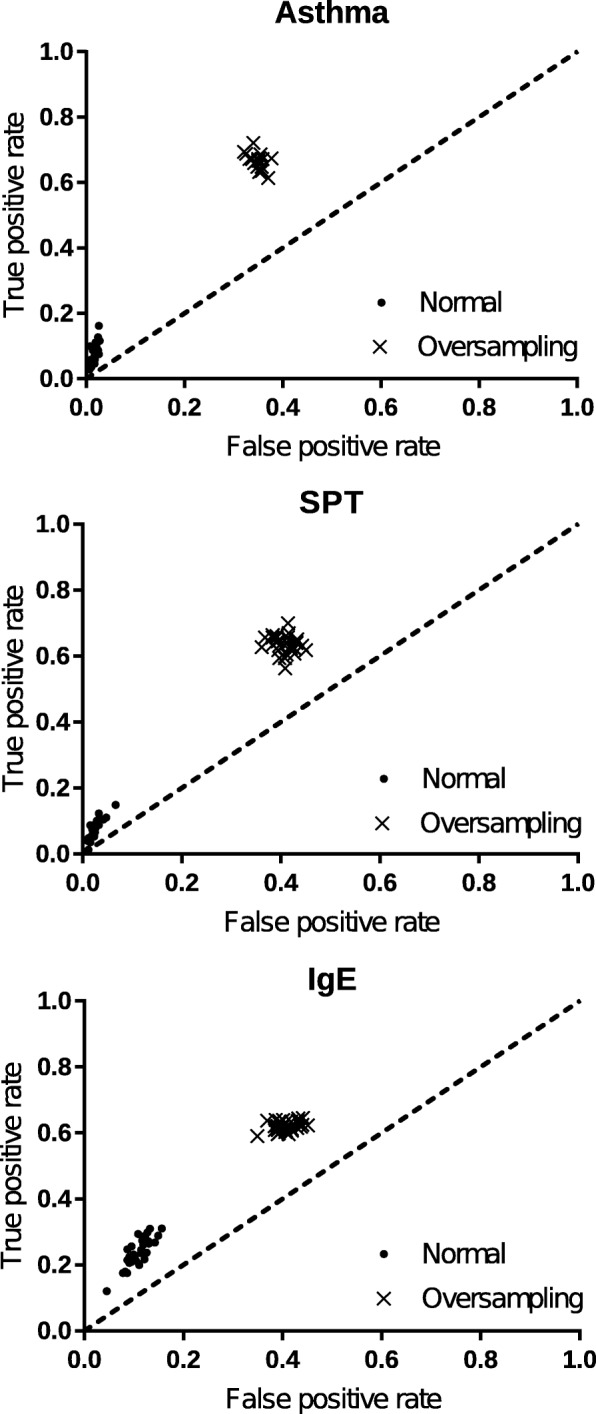
ROC space for training groups of RL algorithm, showing the difference of balanced data and unbalanced data

The variables used in this study and the variable frequencies are shown in Table 1. This population had high prevalence of asthma (22.9*%*), SPT (30.3*%*) and IgE (38.6*%*) positivity. Such high prevalence has as consequence, the number of positive cases approaching the number of negatives cases, so that an unbalanced problem was not expected. However, as shown in Fig. 5, the data balancing had a profound effect on improving the ability to predict positive cases for these conditions, thus balancing type 1 and type 2 errors. Other studies also showed the importance of data balancing in classification algorithms applied to epidemiological problems [7, 68].

**Fig. 6:**
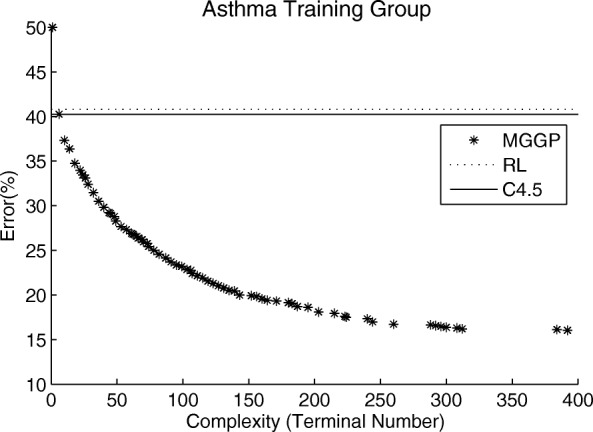
The classification error and the complexity of the set of non-dominated solutions for a training group in the final generation of an MGGP execution. The RL are classification error of RL algorithm for the same training group. C4.5 are the classification error of the same training group in C4.5 algorithm

All executions of MGGP showed a good range of tradeoff between complexity and error. An execution example is displayed in Fig. 6. This shows that the MGGP was able to find a diverse set of optimal solutions, each with different tradeoff between complexity and accuracy. It is evident that for the set of non-dominated solutions be large, it is not possible to generate low complexity solutions with low misclassification, because that would make this solution dominate the other solutions and reduce the size of the non-dominated set. The list with the best models found by MGGP can be downloaded in Additional file 1.

Solutions with low complexity are too simple to explain asthma and allergy and consequently have low accuracy. With increasing complexity the misclassification number tends to drop, however very complex models tend to get very specific to the studied sample and lose the ability to explain other databases. To avoid losing such ability, at the end of execution non-dominated models with respect to the validation group are selected. Despite the best model be the one with the smallest error in the validation group, the solutions with less complexity should not be discarded, as they have the potential to highlight relationships relevant to the understanding of the problem.

**Fig. 7:**
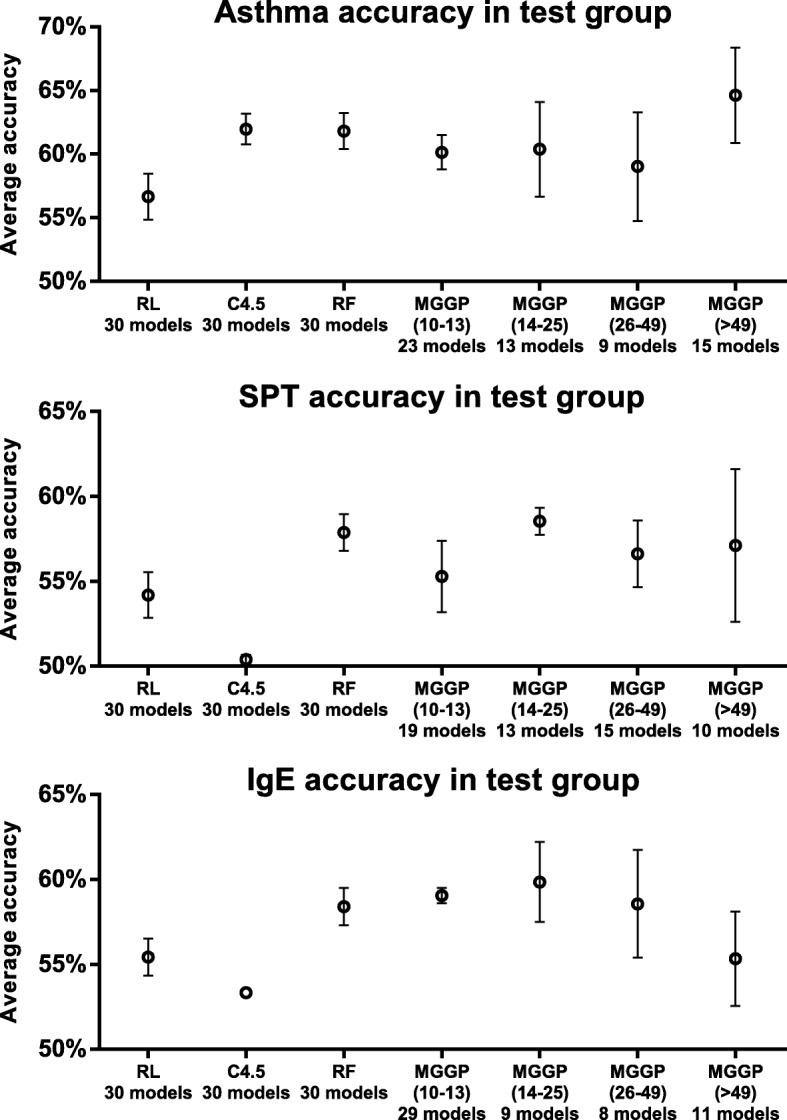
Average accuracy and their 95% confidence interval for solutions of asthma, SPT and IgE in the test group obtained by algorithms RL, C4.5, RF and different ranges of complexity for solutions obtained by MGGP

The set of solutions obtained by MGGP are non-dominated solutions with respect to the validation group obtained at the last generation. To evaluate these solutions the accuracy in the test group was adopted. Table 2 shows the performance of the different techniques evaluated in the test group. The test group is a single group for every 30 runs of the algorithm. Despite the fact that the test group is small (10% presents data), it is composed of data unseen in any execution. So it used to test the general performance of a given solution in different executions. Although most of the best solutions obtained by MGGP showed complexity lower than 50 terminals, a few complex solutions with good accuracy and generalization were found. Each MGGP run took an average of 28.1h on an intel i7 7500 2.7GHz computer with 8GB DDR4 ram. The current version does not have parallelism capability and we expect to have great performance impact when parallelization is implemented in a future release. The average accuracy comparison among RL, C4.5, RF and MGGP with different complexity ranges is shown in Fig. 7. With respect to asthma, RF, C4.5 and MGGP solutions showed no significant differences in average accuracy. However MGGP with complexity between 10 and 13 had significantly greater average accuracy than RL *p*−value=0.003 test T student. With respect to SPT, the RL, RF and MGGP showed no significant differences in accuracy, while C4.5 shows low performance. In IgE outcome, the MGGP with complexities between 10-13 and 14-25 showed higher average accuracy than RL (*p-value*<0.001 and 0.002 respectively test T student) and C4.5.

**Table 2:** Accuracy obtained in the test groups for different techniques, where RL is logistic regression, RF is random forest and * indicates that all executions converged to the same model

Asthma					
	Mean	Median	sd	Min	Max
RL	56.67	56.19	4.82	45.71	63.81
C4.5	61.97	62.86	3.21	55.24	66.67
RF	61.81	62.38	3.78	53.33	68.57
MGGP	61.15	62.36	5.53	50.64	71.87
SPT					
RL	54.19	55.24	3.60	44.76	60.00
C4.5	50.38	50.48	0.72	46.67	51.43
RF	57.87	57.62	2.91	52.38	65.71
MGGP	56.69	57.74	4.18	49.02	66.46
IgE					
RL	55.43	55.24	2.94	50.48	63.81
C4.5 *	53.33	53.33	0	53.33	53.33
RF	58.39	58.49	2.92	52.83	64.15
MGGP	58.39	58.57	3.05	48.39	63.26

## Discussion

Most epidemiological studies use techniques that capture only linear relationships between predictor variables, as for example RL. MGGP for asthma and IgE finds solutions with accuracies better than RL, indicating that there are important complex relationships that RL solutions cannot capture. Although C4.5 and RF was able to find complex solutions, this algorithm showed a lower average accuracy than MGGP.

The RF presented accuracy equivalent to MGGP, but the objective is not to predict asthma and allergy, as this would not be expected based only on studied factors. Because these are complex pathologies with multiples still unknown risk factors. The objective of this work is to find relationships between the studied factors that could potentially be related to asthma and allergies. RF is not useful for that objective because has little capacity to clarify these relations. Another application of computational intelligence in the study of asthma in children, found 62% accuracy using environmental and genetic information [6]. The fact that MGGP achieves equivalent values for asthma using only environmental data, and better results than RL and C4.5, shows the potential of MGGP to discovery relations when applied to complex epidemiological studies.

Examples of relations obtained by MGGP are shown in Table 3. For asthma we note that an important feature that appears in many relationships is the low age. Asthma is a heterogeneous condition with different phenotypes and clinical expressions. A common phenotype of asthma is the transient wheezing phenotype that is not commonly associated with a family history of asthma or with atopy. For this phenotype, the symptoms tend to regress at age 3-5 years old [69], and the high prevalence of this phenotype may explain this relation with low age. Some less complex relationships commonly found were: (i) low age or dog at home are related to asthma, indicating that dog at home is also related with increased asthma, (ii) cat at home or low age increasing asthma, indicating that cat at home is also related with increased asthma (iii) suspected mother psychological disorder also show increased chance to be asthmatic. Some relationships found that affect the chance of being positive for SPT were the presence of infections *T. trichiura* and HSV, with hygiene marker as frequent linen exchange bed and sewage disposal. Other important relation found with SPT, was the high consumption of foods rich in frying (pattern 3) and predominance of sausages, eggs and red meat (pattern 4). This results indicating that those infections, environment, and feeding behavior may influence SPT positivity.

**Table 3:** Examples of relations found by MGGP for IgE, SPT and asthma. The first column is odds ratio of all database without test group, second column is odds ratio in the test group, and the third column is accuracy of relation express. Where “ {1}” means positive for outcome, “ {0}” negative for outcome

Asthma				
All database without test group	Test group			
OR(C.I.95%)	OR(C.I.95%)	Accuracy (%)	Complexity	Important relations
2.42(1.96; 2.99)	1.28(0.69; 2.41)	53.1	10	if((Dog at Home != 0) or (Age = 0)){ 1 }else{ 0 }
2.48(2.01; 3.06)	3.80(2.01; 7.55)	66.0	10	if((Cat at Home = 1) or (Age = 0)){ 1 }else{ 0 }
2.64(2.11; 3.31)	3.25(1.64; 6.42)	63.0	10	if((Mother Psychological disorder = 1) or (Age = 0)){ 1 }else{ 0 }
2.33(1.89; 2.88)	2.36(1.25; 4.43)	60.5	10	if((Dog at Home != 1) and (Mother Psychological disorder = 0)){ 0 }else{ 1 }
3.25(2.62; 4.03)	3.23(1.69; 6.14)	64.2	14	if((Age = 0) or ((Cat at Home = 1) and (Nutritional Factor3 <= 0)))
				{ 1 }else{ 0 }
3.26(2.63; 4.07)	2.92(1.53; 5.56)	63.0	14	if(((Age > 0) or (Nutritional Factor2 = 2)) and
				(Cat at Home != 1)){ 0 }else{ 1 }
3.59(2.87; 4.49)	2.56(1.34; 4.88)	61.1	18	if(((Cat at Home = 1) and (Nutritional Factor3 <= 0)) or
				((Nutritional Factor2 < 2) and (Age = 0))){ 1 }else{ 0 }
3.86(3.10; 4.80)	2.50(1.32; 4.73)	61.1	22	if(((Age != 0) or (((Mother Psychological disorder = 0) and
				(Dog at Home != 1)) and (HZV = 1))) and (Cat at Home != 1)){ 0 }else{ 1 }
3.91(3.12; 4.93)	1.73(0.93; 3.23)	56.8	22	if(((HSV != 1) and ((Linen Bed Exchange != 0) and (Age = 1))) xor
				((Dog at Home != 0) or (Age <= 0))){ 1 }else{ 0 }
4.45(3.57; 5.56)	2.78(1.46; 5.28)	62.3	31	if(Cat at Home = 1){ 1 }else{ (if(((Alumbricoides != 0) or
				(Nutritional Factor2 < 2)) and ((Dog at Home != 0) or (((HZV != 1) or
				(Mother Psychological disorder = 1)) and (Age < 1)))){ 1 }else{ 0 }) }
18.01(13.85; 23.60)	3.77(1.91; 7.46)	64.8	231	too large to show
SPT				
2.03(1.62; 2.55)	2.44(1.22; 4.86)	60.3	10	if((Linen Bed Exchange != 0) and (Ttrichiura != 1)){ 1 }else{ 0 }
2.01(1.61; 2.51)	1.58(0.81 ;3.06)	55.5	10	if((Nutritional Factor4 > 0) and (Linen Bed Exchange != 0)){ 1 }else{ 0 }
1.93(1.55; 2.41)	2.92(1.46; 5.86)	62.3	10	if((Linen Bed Exchange = 0) or (BMI != 0)){ 0 }else{ 1 }
2.11(1.67; 2.66)	1.52(0.77; 2.99)	54.8	10	if((HSV = 0) and (Linen Bed Exchange != 0)){ 1 }else{ 0 }
2.46(1.97; 3.08)	2.06(1.06; 3.98)	58.9	14	if(((HSV = 0) or (Nutritional Factor3 >= 1)) and
				(Linen Bed Exchange != 0)){ 1 }else{ 0 }
2.68(2.15; 3.35)	2.45(1.26; 4.77)	60.9	18	if(((HSV = 0) or (daycare = 1)) and ((Nutritional Factor4 != 1) or
				(Linen Bed Exchange != 0))){ 1 }else{ 0 }
2.45(1.96; 3.07)	2.23(1.14; 4.38)	60.0	20	if((HSV != 0) and (Nutritional Factor3 = 0)){ (if(Nutritional Factor1 != 1)
				{ 0 }else{ 1 }) }else{ (if(Linen Bed Exchange != 0){ 1 }else{ 0 }) }
3.57(2.85; 4.49)	2.31(1.19; 4.48)	60.3	39	if((Nutritional Factor4 < 1) xor (((Nutritional Factor2 >= 1) or
				((num siblings = 1) and (Fly at Home = 0))) and
				(((Mother Psychological disorder != 1) or (num siblings >= 1)) xor
				(Linen Bed Exchange = 0)))){ (if((HSV != 1) or (Tgondi != 1))
				{ 1 }else{ 0 }) }else{ 0 }
6.73(5.30; 8.59)	2.35(1.14; 4.85)	58.9	124	too large to show
IgE				
1.76(1.39; 2.22)	2.28(1.12; 4.63)	60.0	10	if((Gender != 1) or (Cat at Home != 0)){ 0 }else{ 1 }
2.00(1.58; 2.53)	1.87(0.93; 3.75)	57.7	10	if((Gender != 0) and (sewage disposal != 1)){ 1 }else{ 0 }
1.63(1.28; 2.08)	1.81(0.88; 3.72)	56.9	10	if((Nutritional Factor1 != 1) and (Gender != 0)){ 1 }else{ 0 }
2.17(1.71; 2.75)	2.37(1.11; 5.06)	59.2	14	if(((Tgondi = 0) and (Gender != 0)) and (sewage disposal != 1)){ 1 }else{ 0 }
2.02(1.60; 2.57)	1.98(0.99; 3.98)	58.5	14	if(((Gender != 1) and (Cat at Home = 0)) or
				(sewage disposal = 1)){ 0 }else{ 1 }
2.39(1.88; 3.04)	1.75(0.87; 3.52)	56.9	18	if(((Nutritional Factor1 = 1) or (Gender = 1)) and ((sewage disposal = 1) xor
				(Tgondi = 0))){ 1 }else{ 0 }
2.46(1.94; 3.12)	2.13(1.05; 4.31)	59.2	22	if(((Tgondi = 1) or (sewage disposal = 1)) or ((Gender != 1) and
				((Nutritional Factor2 <= 0) xor (sewage disposal = 0)))){ 0 }else{ 1 }
3.64(2.84; 4.69)	2.92(1.43; 5.96)	63.1	46	if((((Tgondi != 1) or ((Nutritional Factor4 = 1) xor
				(paving of the street != 1))) xor (sewage disposal != 1)) or ((Gender != 1) and
				((((Age <= 1) and (Nutritional Factor2 < 1)) xor (sewage disposal = 0)) xor
				(((Nutritional Factor1 <= 0) xor (Ttrichiura = 1)) and
				(num siblings > 2))))){ 0 }else{ 1 }
3.89(3.05; 4.98)	2.14(1.06; 4.35)	59.2	58	if((Tgondi = 0) xor ((Nutritional Factor4 <= 0) and (Age < 2)))
				{ (if(((Gender = 1) xor (Ttrichiura = 1)) or ((sewage disposal != 0) xor
				(Nutritional Factor2 <= 0))){ (if((((BMI = 1) xor (HAV = 0)) or
				(Nutritional Factor1 >= 1)) xor (sewage disposal != 1)){ 0 }else{ 1 }) }else
				{ 0 }) }else{ (if(Gender != 0){ (if(HZV = 0){ 1 }else{ 0 }) }else{ 0 }) }

A variable followed by “ 0” means negative for this variable, and “ 1” is positive

The models generated by MGGP to explain IgE showed that male gender was related to having IgE. The absence of infections such as *T. gondii* and *T. trichiura* as well as sewage disposal are associated with increased IgE. A model that may provide some information for understanding IgE was the one given by: “if(((Nutritional Factor1 = 1) or (Gender = 1)) and ((sewage disposal =1) xor (Tgondi = 0))){ 1 }else{ 0 }”. This model indicates that when a person has moderate levels of consumption of fish, fruit, cereals, or is male, and also shows the absence of *T. gondii* infections or exclusive presence of sewage system, the chance of having IgE increases. Many biological phenomena do not have a linear behavior. Immune cells like lymphocytes, when stimulated have their response increased. However, excess stimulation leads to anergy or apoptosis of these cells, thus reducing the response. This kind of behavior is hardly detected properly using RL. In case of male gender or moderate values of feed pattern 1, it is possible to see in this model and others that both the presence of sewage and the absence of *T. gondii* infection increase the chance of being positive for IgE. This model indicates that excess risk factors may lead to a reduction in the chances of being IgE positive. This type of IgE behavior is reinforced by the frequent occurrence of the “xor” logical operator in more complex models.

We also performed MGGP runs for each outcome on all 1047 individuals without separating by groups. Even knowing that we could not avoid problems of overfiting, we want to observe models that take into account the maximum number of people possible. For asthma we found if((age ¡ =0) or (gat == 1)) 1 else 0, suggesting that low age is important in asthma as discussed earlier. The presence of a cat in the house and its association with asthma has presented contradictory results in literature. Some studies find a positive association with asthma [70, 71]. Others found a negative association [72]. One of the reasons for such disagreements between the works is that the presence of a cat may enhance asthma symptoms, so it is common for parents with asthmatic children to avoid cats, which could cause a negative association in most studies. The list of the best models generated by MGGP in all individuals is shown in the material supplements.

## Conclusion

The use of MGGP can be a good alternative to the understanding of epidemiological problems mainly in the study of complex diseases. Among the qualities presented by this technique, we can highlight:

- MGGP works with classification models and non-linear regression.
- MGGP can generate models with a wide variety of operations such as conditionals (if, else), comparisons (≥, ≤, =, ≠), arithmetic (+, ÷) and specific operations customized to the application domain.
- MGGP makes it possible to define rules to deal with variables of different types such as continuous, discrete, categorical, among others. It is also possible to define how and what operations are possible between the different types of variables.
- MGGP employs rules that restrict the construction of the models, allowing the researcher to add knowledge through the insertion of known relations and the removal of relations that do not make sense.
- MGGP allows the researcher to define groups according to some criteria (such as economic, environmental and nutritional). This type of constraint allows for the definition of different operations upon members of different groups.
- In a single MGGP run, solutions with different levels of complexity can be generated, which improve the understanding of intricate relationships among variables in epidemiological studies.

The use of MGGP performed well compared to RL and C4.5. The application of MGGP in a study focused on asthma and allergies has shown that infections, psychosocial, nutritional, hygiene, and socioeconomic factors may be related in intricate ways with these outcomes. For instance, MGGP showed that the presence of concurrent risk factors for IgE may lead to a reduction in the chances of being IgE positive. This kind of finding could be hardly detected properly using traditional regression based epidemiological techniques.

## Additional file

Additional file 1 List of the best models found by MGGP. List of the best models found by MGGP for Asthma, SPT and IgE. (TXT 112 kb)

## Abbreviations

BMI: body mass index
EBV: Epstein-Baar virus
GNI: gross national income
HSV: Herpes simplex virus
HZV: Herpes zoster virus
HAV: Hepatitis A virus
IgE: Immunoglobulin E antibody
IgG: Immunoglobulin G antibody
MGGP: Multiobjective grammar-based genetic programming

## Footnotes

1 Given a set of symbols V, then V* is the set of all strings over symbols in V or the empty string.

2
http://www.cs.waikato.ac.nz/ml/weka/index.html
